# Influence of Knowledge and Attitude on Lifestyle Practices Among Seventh-Day Adventists in Metro Manila, Philippines

**DOI:** 10.1007/s10943-020-01091-8

**Published:** 2020-10-06

**Authors:** Cesar Augusto Galvez, Chirlynor Calbayan, Kepha Pondi, Maria Vallejos

**Affiliations:** 1 Unidad de Posgrado de Salud Pública, Universidad Peruana Unión, Km 19 Carretera Central, Ñaña, Lurigancho, Lima 15, Peru; 2 Public Health Department, Adventist International Institute of Advanced Studies, Lalaan I, 4118 Silang, Cavite Philippines; 3 Asia Pacific Research Center, Adventist International Institute of Advanced Studies, Lalaan I, 4118 Silang, Cavite Philippines; 4 Centro de Investigación e Innovación en Desarrollo Empresarial, Universidad Peruana Unión, Km 19 Carretera Central, Ñaña, Lurigancho, Lima 15, Peru

**Keywords:** Adventist Health Message, Attitudes, Knowledge, Lifestyle practices

## Abstract

This cross-sectional study examined the influence of knowledge and attitude on lifestyle practices (KAP) of the five dimensions of the Adventist Health Message (AHM5D). A sample of 1442 respondents was drawn from seven Seventh-day Adventist Churches in Metro Manila, Philippines. Hierarchical multiple regression showed that the social dimension of knowledge and the physical, spiritual, and social dimensions of attitude, significantly influenced the practices of AHM5D (*β* = − .056, *p* = .037; *β* = .236, *p* < .001; *β* = .211, *p* < .001; *β* = .145, *p* < .001, respectively), with *r*^2^ = .334. These findings suggest more effective interventions in the AHM5D promotion.

## Introduction

Lifestyle-related disease complications are the leading causes of death around the world. Non-communicable diseases (NCD) account for about 40 million (70%) deaths around the world (WHO 2017). In the Philippines, NCD accounted for 57.3% of all deaths (WHO 2014). Paradoxically, this death toll can be prevented through healthy lifestyle practices such as physical activity, healthy diet, and avoidance of alcohol consumption and tobacco use, which are part of the Seventh-day Adventist (SDA) lifestyle (Sharma et al. 2017).

The Seventh-day Adventist (SDA) Church advocates a healthy lifestyle around the world. The SDAs believe that “health reform and teaching of health and temperance are inseparable parts of the church’s message” (General Conference of the Seventh-day Adventist 2010, p. 140). Studies in developed countries, particularly in North America, Europe, and Oceania, showed a significant health advantage in SDAs when compared with the rest of the population (Butler et al. 2008; Fraser 2003; Kent and Worsley 2009; Rizzo et al. 2011; Taylor 2013; Thygesen et al. 2012). SDAs from California have a lower risk of cancer, cardiovascular disease, and ischemic heart diseases related to lifestyle (Mills et al. 1994). Furthermore, SDA men live 7.3 years, and women 4.4 years longer than their non-SDA peers (Fraser and Shavlik 2001). Religious involvement was also found to be associated with positive lifestyle practices among Black SDAs in Canada (McKenzie et al. 2015). SDAs from Denmark in the Czech Republic showed decreased risks of cancers and cardiovascular diseases when compared with the rest of the population (Slavícek et al. 2008). In addition, Australian SDA adolescents showed better scores on markers of cardiovascular health than their peers (Grant et al. 2008).

Studies in developing countries regarding the health and lifestyle of SDA members are limited. A study of lifestyle intervention among 7172 SDAs and non-SDAs in North America found a reduction in chronic disease risk factors associated with lifestyle practices (Kent et al. 2016). In the Caribbean region, vegetarian SDAs showed lower rates of circulatory diseases, cancer, and type 2 diabetes than omnivorous groups (Li 2014). Furthermore, in the Philippines, a study found a relationship between stress-related problems, diet, and physical activity among the SDA Church employees (Segovia-Siapco 2014).

Although SDAs emphasize the promotion of a healthy lifestyle as part of their mission and have been found to have better health and longer life than their peers, it is unknown how much SDA Church members know about the Adventist Health Message-Five Dimensions (AHM5D) namely; the physical, mental, spiritual, social, and environmental dimensions. In addition, the level of influence that knowledge and attitude exert toward lifestyle practices with respect to the AHM5D among SDAs in developing countries remains largely unknown.

The AHM5D is Biblically founded. According to Biblical anthropology, man is a whole biopsychosocioecospiritual being, a living soul which is expressed holistically in physical, mental, spiritual, social, and environmental dimensions (Galvez 2014). This framework suggests that whatever affects one dimension of health affects the other dimensions, too. Ellen White, a founder of the SDA Church, presented interrelations of the different dimensions of health; “since the mind and the soul find expression through the body, both mental and spiritual vigor are in great degree dependent upon physical strength and activity; whatever promotes physical health, promotes the development of a strong mind and a well-balanced character” (White 1905, p. 195). Science also has corroborated the wholeness of human nature. For instance, every thought and feeling has an electrical record in the brain and produces a biochemical reaction through the neurotransmitters (Taylor 2013). Furthermore, focus on spirituality is likely to improve “person-centered approaches to wellbeing long sought by patients and clinicians” (VanderWeele et al. 2017, p. 519).

**Fig. 1:**
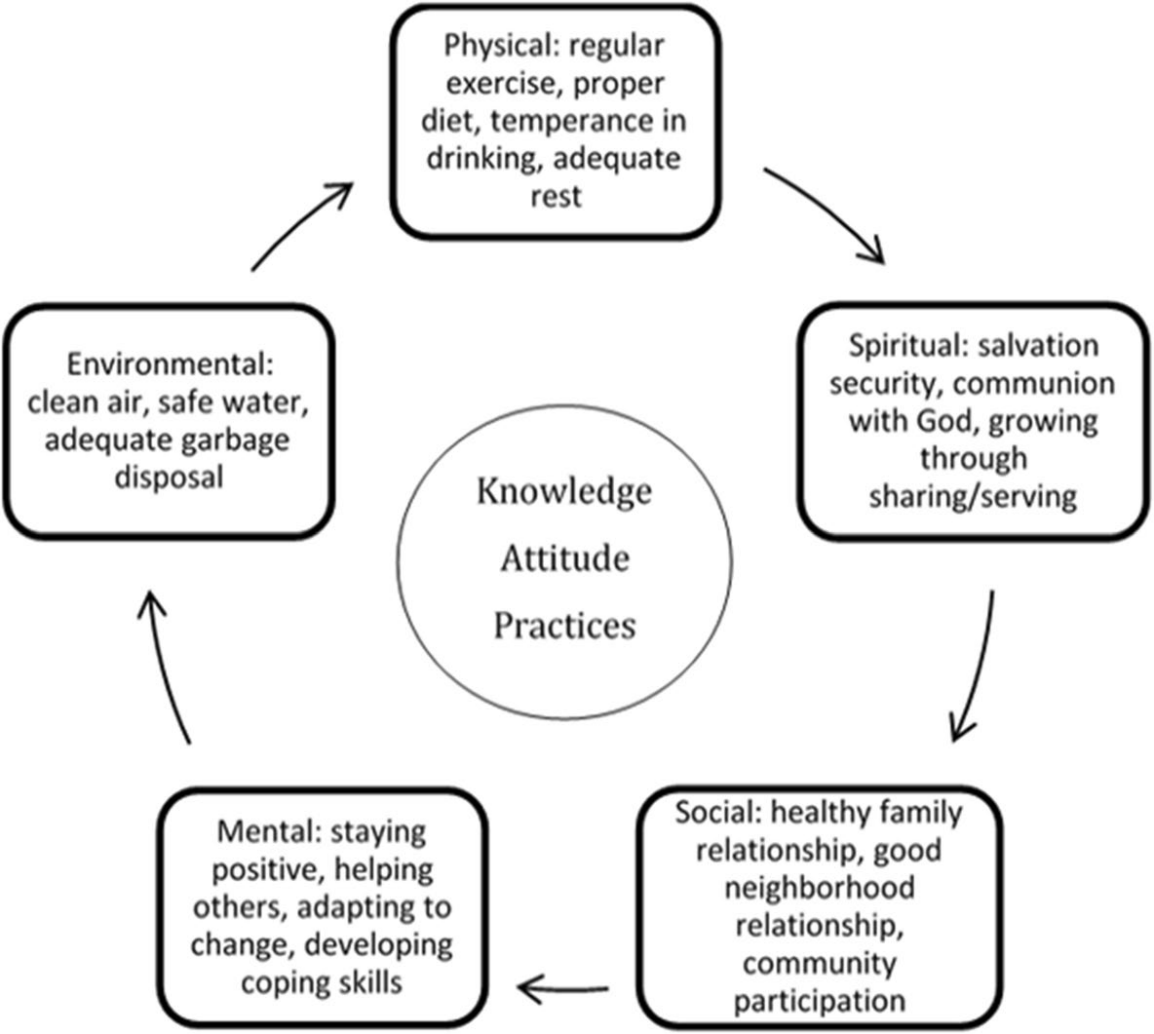
The Adventist Health Message’s five dimensions

The selected major components for each of the five dimensions in this study were: the physical dimension includes regular exercise, proper diet, temperance in drinking, and adequate sleep/rest; the mental dimension includes staying positive, helping others, adapting to change, and developing coping skills; the spiritual dimension includes salvation security, communion with God, and growing through sharing/serving; the social dimension includes healthy family relationships, good neighborhood relationship, and community participation; and the environmental dimension includes clean air, safe water, and adequate garbage disposal (see Fig. 1).

This study therefore sought to determine the influence of knowledge and attitude in terms of physical, mental, spiritual, social, and environmental health dimensions on lifestyle practices among Filipino SDA Church members in Metro Manila. This study contributes to the body of knowledge. Moreover, the results of this study may be useful in planning interventions and improving healthy lifestyle promotion strategies to reduce the incidences and prevalence of NCDs.

## Methods

This cross-sectional survey was conducted on both males and females from the seven largest SDA churches in Metro Manila, Philippines. Ethical consideration approval (ERB/2016-26) was obtained from the Ethics Review Board (ERB) of the Adventist International Institute of Advanced Studies (AIIAS). In addition, permission for data collection was secured from the Central Luzon Conference, a territory of North Philippine Union Conference of the SDA Church, and leaders of the churches. Furthermore, informed consent for participation in the study from the respondents was sought before the commencement of the study.

The questionnaire for collecting the data was constructed based on the AHM5D which is comprised of the physical, spiritual, social, mental, and environmental health dimensions to evaluate the level of knowledge, attitude, and lifestyle practices of SDAs in Manila. A team of five experts, following a Delphi design validated the questionnaire. A pilot study on respondents was also conducted at three churches located in the Cavite province on the southern shores of Manila Bay. The reliability test using Cronbach’s alpha was .63 for knowledge, .75 for attitude, and .83 for practice.

The final self-administered questionnaire, which consisted of 103 questions were answered within approximately 30 min. The questionnaire was comprised of four sections. The first section consisted of 25 questions about knowledge of the AHM5D in its five dimensions; physical, spiritual, social, mental, and environmental health. Most of the questions were multiple choice. The second section consisted of 27 questions on attitude toward the AHM5D. The questions used 5-point Likert response categories. The third section consisted of 43 questions on practices about physical habits, spiritual life, social relations, mental health, and environmental care. Most of the questions were multiple choice. Finally, the section on personal information included sex, age, educational level, monthly income, number of household members, region of origin, years of being SDA, and being born in an SDA family.

Trained AIIAS research assistants did the data collection. A total of 1900 questionnaires were administered to the respondents from the seven selected churches. However, there were only 1442 respondents included in the study after 458 cases were excluded due to incomplete information or duplicate answers. The data collected were entered and analyzed using the SPSS 22.0 software package. After cleaning the data, AHM5D for knowledge, attitude, and practices, and their dimensions (physical, spiritual, social, mental, and environmental health) were calculated to get the total sum of the scores of each section (see Table 1). A score of one point was allocated for a correct response in knowledge, while a zero score was allocated for a “don’t know” answer or a wrong answer. The maximum overall knowledge score was 25 points. A verbal interpretation of “poor” was given to scores less than 15, “average” was given to 15–20, and “good” was given to more than 20 up to 25 points. The categories of the rest of the dimensions (physical, spiritual, social, mental, and environmental) were based on the sum of the scores of each section.

**Table 1:** Scoring criteria for knowledge, attitude, and practices

	Knowledge			Attitude			Practices		
	Poor	Average	Good	Negative	Neutral	Positive	Poor	Average	Good
Overall	< 15	15–20	20.1–25	< 81	81–108	108.1–135	< 60	60–80	80.1–100
Physical	< 3.6	3.6–4.8	4.9–6	< 18	18–24	24.1–30	< 32.4	32.4–43.2	43.3–54
Mental	< 3.0	3.0–4.0	4.1–5	< 15	15–20	20.1–25	< 3.0	3.0–4.0	4.1–5
Spiritual	< 3.6	3.6–4.8	4.9–6	< 18	18–24	24.1–30	< 8.4	8.4–11.2	11.3–14
Social	< 2.4	2.4–3.2	3.3–4	< 15	15–20	20.1–25	< 9.0	9.0–12.0	12.1–15
Environmental	< 2.4	2.4–3.2	3.3–4	< 15	15–20	20.1–25	< 7.2	7.2–9.6	9.7–12

AHM5D attitude responses were computed on a Likert scale (1–5). The 11 negative statements were inverse scored, and all positive statements were recorded; the maximum overall score was 135 points. Similarly, attitude was categorized as negative, neutral, and positive based on the sum of the scores of each question overall and for each dimension.

Since AHM5D practices were measured using a five-point Likert scale and multiple choice questions, scores with higher points were allocated to the most favorable practice and lower points allocated to the undesirable practices. The nine negative statements were inverse scored, and all the positive statements were recorded. The maximum score was 100 points. Also, practice was categorized by poor, average, and good based on the sum of the scores of each question overall and for each dimension. Finally, descriptive statistics, Chi-Square test of independence, correlations, and hierarchical multiple regression analyses were used where appropriate, with the level of significance set at *p* < .05.

## Results

### Sociodemographic Characteristics

Among the 1442 respondents, 853 were females (59.2%) and 544 were males (37.7%). Forty-five respondents did not indicate their gender. More than half of the respondents (55.7%, *n* = 803) were between 18 and 35 years old. Most of the participants (70%, *n* = 1010) had completed college, and 46.1% (*n* = 665) received a monthly income between 5000 and 40,000 Philippine pesos. Those who indicated that they had two to five persons living in their homes were 885 (61.4%). Further, 68.5% came from Luzon (*n* = 988), 14.6% from Visayas (*n* = 211), and 14.4% from Mindanao (*n* = 207). 2.5% did not provide information on their origin. 23% (*n* = 332) of the respondents reported to have been members of the SDA church for less than 10 years, 23.3% (*n* = 336) have been members between 11 and 20 years, and 50.6% (*n* = 730) have been members for more than 20 years. Around three percent (3.1%) (*n* = 44) did not provide information about how long they have been members of the SDA church. In addition, 63.5% (*n *=915) reported to have been born in an SDA family and 34.3% (*n *=495) of them were not born in an SDA family. 2.2% of the respondents did not provide information on whether they were born in an SDA family or not.

#### Knowledge of the Adventist Health Message

Table 2 shows 23.4% of the respondents have poor knowledge, 71.7% have average knowledge, and 4.9% of the respondents have good knowledge of the AHM5D. In the environmental dimension of knowledge, the scores were mostly poor (97.8%). In the physical dimension, the scores were between poor and average (52.4% and 47.4%, respectively). However, the majority (93.3%) indicated that having a vegetarian diet is healthy and nutritionally adequate. Moreover, more than half of the respondents (60.5%) indicated that meats are not necessary for a healthy diet. In contrast, only 0.3% indicated that moderate exercise for at least 30 min 5 days a week is necessary. Additionally, only 0.3% indicated adults need to sleep between 7 and 9 h per day.

**Table 2:** Level of knowledge, attitude, and practices of the respondents

KAP	*n* (%)	*n* (%)	*n* (%)	*M* (SD)	Categorical interpretation
Knowledge	Poor	Average	Good		
Overall	338 (23.4)	1034 (71.7)	70 (4.9)	16.70 (2.23)	Average
Physical	755 (52.4)	683 (47.4)	4 (0.3)	3.34 (0.75)	Poor
Mental	493 (34.2)	34 (2.4)	915 (63.5)	3.54 (0.72)	Average
Spiritual	173 (12.0)	252 (17.5)	1017 (70.5)	5.03 (1.08)	Good
Social	59 (4.1)	313 (21.7)	1070 (74.2)	3.69 (0.57)	Good
Environmental	1410 (97.8)	31 (2.1)	1 (0.1)	1.09 (0.73)	Poor
Attitude	Negative	Neutral	Positive		
Overall	11 (0.8)	710 (49.2)	721 (50.0)	107.48 (10.40)	Neutral
Physical	47 (3.3)	579 (40.2)	816 (56.6)	24.06 (3.03)	Positive
Mental	47 (3.3)	393 (27.3)	1002 (69.5)	20.64 (2.59)	Positive
Spiritual	31 (2.1)	474 (32.9)	937 (65.0)	24.80 (3.06)	Positive
Social	55 (3.8)	497 (34.5)	890 (61.7)	20.09 (2.47)	Positive
Environmental	313 (21.7)	618 (42.9)	511 (35.4)	17.90 (3.76)	Neutral
Practice	Poor	Average	Good		
Overall	450 (31.2)	848 (58.8)	144 (10.0)	65.65 (10.73)	Average
Physical	869 (60.3)	511 (35.4)	62 (4.3)	30.78 (7.25)	Poor
Mental	510 (35.4)	21 (1.5)	911 (63.2)	3.64 (1.22)	Average
Spiritual	718 (49.8)	498 (34.5)	226 (15.7)	8.44 (2.76)	Average
Social	213 (14.8)	280 (19.4)	949 (65.8)	12.12 (2.55)	Good
Environmental	48 (3.3)	233 (16.2)	1161 (80.5)	10.67 (1.46)	Good

In the mental dimension, 63.5% fell in the scoring category of good. 89.2% of the respondents indicated that helping others is good for mental health, while 83.3% indicated that dwelling on negative experiences is harmful to mental health. In the spiritual dimension, 70.6% of respondents had good knowledge. Moreover, the majority of the respondents (97.4%) indicated that personal prayer is vital for communion with God. In addition, 78.9% indicated that studying the Bible is vital for communion with God. In the social dimension, 74.2% showed good knowledge, while 97% answered that a loving and respectful relationship between spouses is important to good health, and that supportive family and friends help in building self-esteem.

#### Attitude Toward the Adventist Health Message

Findings on attitude toward AHM5D show that 0.8% of the respondents had negative, 49.2% had neutral, and 50% had positive attitude. On the environmental dimension of attitude, scores were mostly neutral (42.9%), with 35.4% positive, and 21.7% negative. Regarding the physical dimension, the majority of the respondents ranged between neutral and positive attitude (40.2% and 56.6%, respectively).

The results on the social dimension showed that the majority (61.7%) had a positive attitude, while (34.5%) were neutral. On the mental dimension, the results showed that the majority (69.5%) of the respondents had a positive attitude. On a similar vein, the results for the spiritual dimension showed that 65% had a positive attitude. 96.2% indicated that regular prayer is vital for communion with God. Furthermore, regarding Bible study, 55.3% of the respondents indicated that Bible study is not optional for developing a growing spiritual life.

#### Practices of the Adventist Health Message

Regarding the practices of the AHM5D, 58.8% of the respondents reported average practices, and 10% reported good practices, while 31.2% reflected poor practices. In terms of the spiritual dimension, the practice scores were mostly between poor and average (49.8% and 34.5%, respectively), and 15.7% indicate good practices. 65.4% had personal prayer more than twice a day, and only 23.7% read the Bible five to seven times per week. In relation to the social dimension, the practice scores were mostly good (65.8%). While, in terms of the mental dimension, the practice scores were mostly good (63.2%).

In relation to the physical dimension, the practice scores were mostly between poor and average (60.3% and 35.4%, respectively). Among the respondents, 94.8% of the respondents never eat pork, 89.7% never eat sea-foods, 68.6% never eat internal organs of animals, 66% never eat goat’s meat, 22.3% never eat beef, 14.4% never eat chicken, and 7.3% never eat fish. In addition to eating plant foods, 83% consume milk and 94.8% consume eggs at least once a week. The consumption of empty carbohydrates ranged between 73.5 and 91.8% at least once a week. The majority (88.8%) of the respondents also indicated that they never drink alcoholic beverages. Moreover, only 26.6% of the respondents do exercise at least 30 min every day. Regarding sleep, only 34.9% sleep 7–9 h every day. In terms of the environmental dimension, the practice scores were mostly good (80.5%).

#### Association Between Knowledge, Attitude, and Practices of AHM5D and the Sociodemographic Characteristics

The association between knowledge, attitude, and practices of AHM5D, and the sociodemographic characteristics of the respondents were analyzed using Chi-Square (see Table 3). The knowledge, attitude, and practices were statistically significant (*p* < .05) for the level of education, monthly income, and number of years as an SDA church member. Gender was significant (*p* < .05) for knowledge and attitude. Furthermore, age was significant (*p* < .05) for knowledge and practices. Additionally, being born in an SDA family was statistically significant (*p* < .05) for practices.

**Table 3:** Association between knowledge, attitude, and practices of the Adventist Health Message and the sociodemographic characteristics of the respondents

Sociodemographic characteristics	Knowledge		Attitude		Practices	
	*X*2	*p* value	*X*2	*p* value	*X*2	*p* value
Gender	6.753	.034	8.195	.017	0.957	.620
Age	21.554	.018	18.085	.054	162.281	.000
Level of education	97.617	.000	63.201	.000	25.681	.001
Income	54.468	.000	72.313	.000	53.220	.000
Number of household	8.111	.423	9.744	.283	13.021	.111
Region	4.362	.359	4.354	.360	4.079	.395
Years of SDA	58.133	.000	35.158	.001	90.180	.000
Born	0.772	.680	1.014	.602	8.702	.013

The females showed better knowledge and attitude than the males. Regarding age, those between 26 and 55 had better knowledge compared to those below 26 and those above 55 years old. However, those from 46 years of age and above had better practices toward AHM5D. In addition, those with at least a college degree showed better knowledge, attitude, and practices. The results also indicated that those with monthly income above 10,000 pesos showed better knowledge, attitude, and practices. Furthermore, those who had been members of SDA church for more than 21 years showed better knowledge, attitude and practices. It was interesting to note that those who were not born in an SDA family had better practices toward AHM5D.

The results showed that there is a significant positive weak correlation between overall knowledge and overall practices of AHM5D (*r* = .230, *p* < .01). Knowledge of physical, mental, spiritual, and social dimensions, and the overall practice of the AHM5D were significant (*r* = .222, .094, .238, and .118, *p* < .01, respectively). In the same vein, the overall attitude and overall practices of AHM5D had a moderate significant correlation (*r* = .416, *p* < .01), while the dimensions of the attitude, physical, mental, spiritual, social and environmental, and practices of AHM5D (*r* = .391, .172, .417, .334, and .158, *p* < .01, respectively) were significant and ranged from weak to moderate correlations.

#### Hierarchical Multiple Regression

Hierarchical multiple regression was used to assess the ability of the five dimensions of knowledge and attitude to predict the practices of AHM5D (see Table 4). The influence of social demographic characteristics of the respondents was controlled. Gender, age, education, monthly income, and years of being an SDA were entered at Step 1 and explained 15.3% of the variance in practices of AHM5D. After the entry of dimensions of knowledge and attitudes at Step 2, the total variance explained by the final model was 33.4%, [*F*(16, 1260) = 39.534, *p* < .001]. This means the AHM5D dimensions of knowledge and attitudes explained the additional 18.1% of the variance in practices of AHM5D, after controlling for gender, age, education, monthly income, and years as an SDA [*r*^2^ = .334, *F*(10, 1260) = 34.282, *p* < .001]. In the final model, the following dimensions were statistically significant with the practices of AHM5D: the social dimension of knowledge (*β* = − .056, *p* < .05), physical dimension of attitude (*β* = .236, *p* < .001), spiritual dimension of attitude (*β* = .211, *p* < .001), and social dimension of attitude (*β* = .145, *p* < .001).

**Table 4:** Hierarchical multiple regression

Model	*b*	SE-*b*	*Beta*	*t*	*p* value
(Constant)	50.950	1.950		26.125	.000
Gender	.613	.567	.028	1.081	.280
Age	2.439	.238	.336	10.238	.000
Education	1.593	.429	.103	3.716	.000
Monthly income	.151	.145	.030	1.044	.297
Years of SDA	.102	.183	.020	.557	.578
Born in SDA family	1.299	.695	.058	1.868	.062
(Constant)	12.446	3.210		3.878	.000
Gender	.518	.508	.024	1.020	.308
Age	2.081	.216	.287	9.621	.000
Education	.761	.389	.049	1.954	.051
Monthly income	− .020	.131	− .004	− .153	.879
Years of SDA	.003	.164	.001	.020	.984
Born in SDA family	.985	.623	.044	1.582	.114
K-Physical	.648	.361	.045	1.794	.073
K-Mental	− .150	.375	− .010	− .400	.689
K-Spiritual	.284	.287	.029	.986	.324
K-Social	− 1.048	.502	− .056	− 2.089	.037
K-Environmental	− .042	.341	− .003	− .124	.901
A-Physical	.833	.100	.236	8.329	.000
A-Mental	− .222	.118	− .054	− 1.886	.060
A-Spiritual	.738	.110	.211	6.684	.000
A-Social	.636	.120	.145	5.296	.000
A-Environmental	− .119	.073	− .043	− 1.638	.102

The dependent variable was practice

Model 1. *r* = .391, *r*^*2*^= .153, Adjusted *r*^*2*^= .149

Model 2. *r* = .578, *r*^*2*^= .334, Adjusted *r*^*2*^= .326

## Discussions

This study sought to determine the influence of knowledge and attitude on SDAs lifestyle in its five dimensions: the physical, mental, spiritual, social, and environmental dimensions. The results showed that the SDA Church members among the population of study have an average knowledge of the SDA health message. However, in a closer analysis, the majority (97.8%) showed poor knowledge in the environmental aspect of the health message. In addition, only .3% indicated that moderate physical exercise for at least 30 min 5 days per week is necessary, and only .3% indicated the adults need to sleep between 7 and 9 per day. These findings stand in contrast to other studies from developed countries among the general population which showed high knowledge in physical activity (Knox et al. 2013). This level of knowledge may be attributed to inadequate teaching and learning about the AHM5D, or to a teaching plan of the AHM5D which may be just focused on diet, without a balance of teaching on other physical dimensions (Galvez 2014).

Although there are studies that indicate good knowledge of healthy lifestyle (Hassan et al. 2015), this study showed relatively average knowledge toward healthy lifestyle practices. Poor knowledge of health is associated with poor health (Mõttus et al. 2014). Consequently, poor knowledge, particularly in the physical dimension of AHM5D, may cause an increase in NCDs among the SDAs in Metro Manila. This finding is not unique to the SDAs under this study, but reflects what was found in other studies among the general public, especially in developing countries (Amarasekara et al. 2016).

The results also showed poor knowledge (97.8%) of the environment dimension of AHM5D, but 35.4% indicated a positive attitude toward the same. The majority (80.5%), however, indicated they practiced the environmental dimension of AHM5D. This disparity may be explained by the enactment of the environmental laws that govern environmental practices in the Philippines that require public involvement, and participation in environmental matters (Gera 2016). On the other hand, the poor knowledge on physical exercise and required amount of sleep may be attributed to the busy city lifestyle which leave people with limited time for both exercise and sleep (Galvez 2016).

The result of knowledge of AHM5D seems to be mirrored in overall practices of AHM5D; poor (31.2%), average (58.8%), and only 10% good. Whereas 50% of respondents indicated positive attitude, only 10% indicated they practiced the AHM5D. These percentages are, however, relatively low compared to other similar studies within the region, which indicate between moderate, and high knowledge on lifestyle practices (Amarasekara et al. 2016; Hassan et al. 2015).

These findings may suggest that although the SDA Church in Manila teaches the AHM5D, the dissemination of the knowledge may not be done effectively, thereby resulting in only average knowledge of AHM5D among the SDA Church members (Galvez 2016). As a result, only some of the SDA Church members may effectively use that knowledge. This is in line with other similar studies that indicate that in spite of health literacy, people do not put the knowledge into practice for health improvement (Hassan et al. 2015).

The results further showed that females indicated more positive attitude and better practices of AHM5D than their male counterparts. This suggests that females are more conscious about their health choices than males; hence, the females opt for healthier lifestyle practices. Similar findings were reflected in other studies (Von Bothmer and Fridlund 2005).

In the final model, age, education, knowledge of the physical dimension, and attitude on the physical, spiritual and social dimensions of AHM5D significantly influenced AHM5D practices, and accounted for 33.4% of the variance. These findings may suggest that there may be other possible factors that account for the remaining 66.6% which may contribute to practices of the AHM5D that are not included in this study. Such factors may be highly personal. Sallis et al. (2000) asserts that an individual’s environment and knowledge about the nature of physical activity and their benefits may make the outcomes of health promotion varied and complex. This finding, however, opens a door for future studies.

In conclusion, the respondents indicated an average knowledge and practice, and 50% positive attitude toward AHM5D. In addition, there was a significant positive relationship between knowledge, attitude, practices, and the sociodemographic characteristics. Females indicated more positive attitude and better practices of AHM5D than males. Finally, the dimensions that predicted practice of the AHM5D are knowledge and attitude of the physical dimension, and attitude of the spiritual and social dimension. Therefore, any health promotional strategies should focus on these dimensions which significantly influence the practices. In addition, different approaches may be needed for the males to develop a positive attitude and good AHM5D practices.
